# Incidental left main bronchus obstruction during left-sided double-lumen tube intubation of a patient with an unrecognized tracheal bronchus: A case report

**DOI:** 10.1097/MD.0000000000005674

**Published:** 2016-12-30

**Authors:** Ho Bum Cho, Hyoung June Kim, Hyung Youn Gong, Mun Gyu Kim, Sang Ho Kim

**Affiliations:** a Department of Anesthesiology and Pain Medicine, Soonchunhyang University Hospital Seoul, Seoul, Korea; b Department of Anesthesiology and Pain Medicine, Soonchunhyang University Hospital Cheonan, Cheonan, Chungcheongnam-do, Korea.

**Keywords:** double lumen tube, fiberoptic bronchoscopy, tracheal bronchus

## Abstract

**Introduction:**

Tracheal bronchus is a right-sided anomalous bronchus arising from the trachea above the main carina and occurs in 0.1% to 2% of the general population.

**Case presentation:**

We present a case of left main bronchus obstruction during a left-sided double-lumen tube intubation in a patient with an unrecognized tracheal bronchus. After the intubation, to confirm the position of the tube, we observed what we believed was the carina with a fiberoptic bronchoscope, but it was a site between the tracheal bronchus and the right main bronchus. Thus, a right-sided intubation was performed, and the left main bronchus was obstructed with a bronchial cuff. As a result of the inappropriate ventilation, peak inspiratory pressure was elevated and arterial oxygen saturation decreased.

**Conclusion:**

Anesthesiologists should keep in mind the possibility of anatomical variation in the large airways, and bronchoscopy should be accompanied by cautious auscultation and confirmation of the division of the bronchus.

## 1 Introduction

Congenital abnormalities of the large airways are not common but can occasionally pose significant difficulties for the anesthesiologist.^[1]^ Tracheal bronchus, that is, a right-sided bronchus arising from the trachea above the main carina, occurs in 0.1% to 2% of the general population.^[2]^ Most patients with a tracheal bronchus are asymptomatic but there have also been reports of various pulmonary symptoms.^[3]^

In cases with an undiagnosed tracheal bronchus, several previous studies have described events in which a single lumen endotracheal tube was obstructed or migrated into a tracheal bronchus accidentally, causing right upper lung atelectasis or inadequate ventilation, ultimately leading to hypoxemia. The same situation can occur while using a double-lumen tube or bronchial blocker.

Here, we present a case of an adult patient with an unrecognized tracheal bronchus that caused mislocation of a double-lumen tube for wedge resection by video-assisted thoracoscopic surgery (VATS).

## 2 Case report

A 32-year-old male, weighing 80 kg with a height of 172 cm, classified as American Society of Anesthesiologists physical status I, was admitted due to sudden dyspnea. He had undergone a chest radiograph and chest computed tomography (CT) at the emergency room. He was diagnosed with spontaneous pneumothorax in the right hemithorax, and then a closed thoracostomy was performed. On the next day, he was scheduled for elective surgery for wedge resection by VATS.

Departing for the operating theater, glycopyrrolate 0.02 mg was given intramuscularly. On arrival in theater, standard monitoring devices including electrocardiography, pulse oximetry, and an oscillometric noninvasive blood-pressure cuff were applied.

General anesthesia was induced using intravenous lidocaine 40 mg, fentanyl 50 μg, propofol 120 mg (1.5 mg/kg), and rocuronium 40 mg (0.5 mg/kg) for neuromuscular blockade. Tracheal intubation was performed with a 37-Fr left-sided double-lumen tube (Mallinckrodt; Covidien, Ireland) using a curved laryngoscope (Macintosh blade 3) and the insertion depth was 30 cm from the upper incisors. Anesthesia was maintained with oxygen, medical air, and 1 minimum alveolar concentration of desflurane.

After intubation, the location of the carina was assessed with a fiberoptic bronchoscope (LF-GP, Olympus Optical Co.; Tokyo, Japan) through the tracheal lumen, and the tube was fixed. Subsequently, both lung fields were auscultated using a stethoscope. We found no sounds in the left lung field and felt high resistance upon manual ventilation. The ventilator was set in volume-controlled mode at a tidal volume of 500 mL with a peak inspiratory pressure (PIP) of 38 cmH_2_O and arterial oxygen saturation (SaO_2_) fell gradually to 92%. We performed a fiberoptic bronchoscopic examination immediately. After pulling the double-lumen tube backwards, by about 2 cm under fiberoptic bronchoscopy, we found the true carina and a tracheal bronchus originating from the trachea.

**Figure 1:**
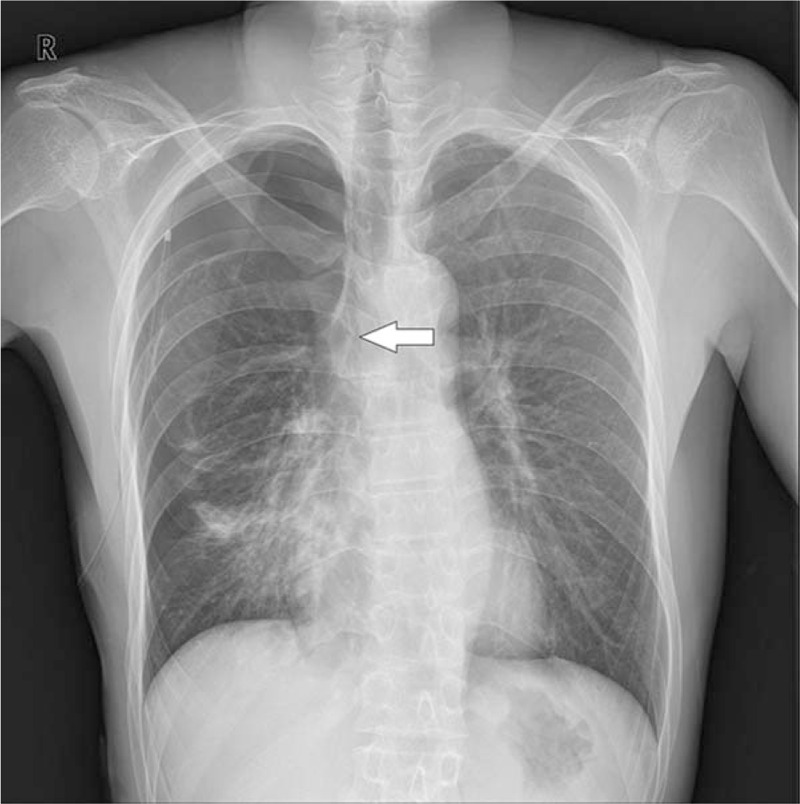
Preoperative chest radiograph shows tracheal bronchus in the right upper lobe with right spontaneous pneumothorax treated with a closed thoracostomy (arrow).

**Figure 2:**
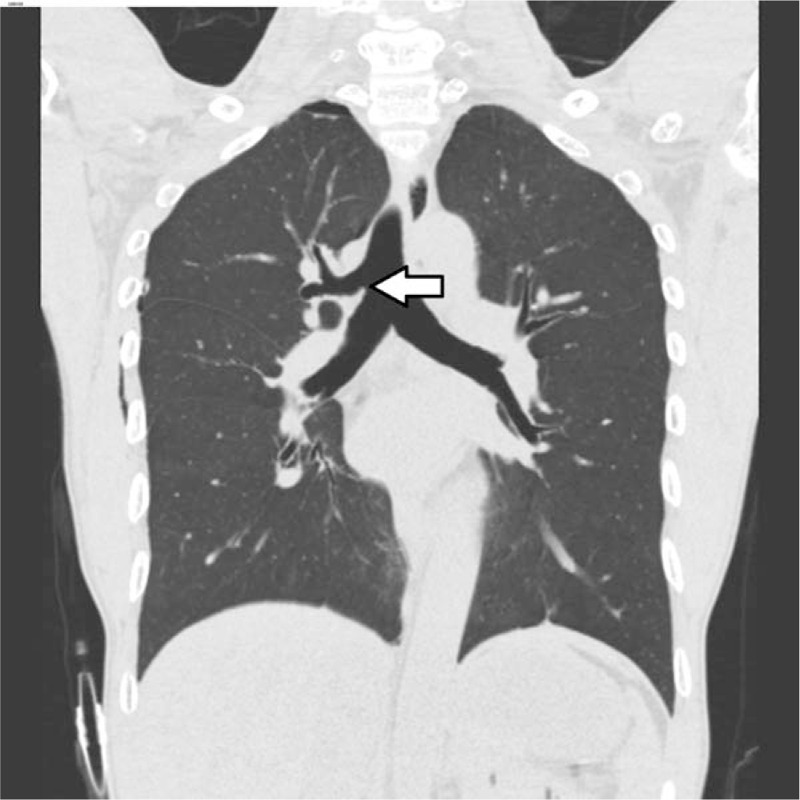
Preoperative chest computed tomography demonstrates a tracheal bronchus supplying the right upper lobe and originating from the right lateral tracheal wall, 1.8 cm proximal to the carina on the coronal reconstructed image (arrow).

Once the double-lumen tube was located correctly, the sounds of both lungs were detected with a stethoscope, and right lung collapse was achieved by tracheal lumen clamping. SaO_2_ was maintained at 98% to 99% with a FiO_2_ of 0.5. We recognized a tracheal bronchus on a retrospective review of the preoperative chest radiograph (Fig. 1) and chest CT (Fig. 2). The total anesthesia time was 80 minutes, and no intraoperative or postoperative complications occurred.

## 3 Discussion

The lung isolation technique is commonly used in thoracic surgeries for safety and to create clarity in the surgical field. One-lung ventilation is usually achieved with a double-lumen endotracheal tube or a bronchial blocker.^[4]^ The choice of device for lung isolation remains controversial. The double-lumen tube has several disadvantages including difficult insertion, profound secretions, and laryngeal trauma.^[5]^ However, it provides high-quality lung isolation.^[6]^

The right upper lobe bronchus normally arises from 1 to 3 cm distal to the carina. However, a tracheal bronchus supplying the right upper lobe usually arises proximal to the carina, within 2 cm.^[7]^ Thus, when using conventional endotracheal intubation without bronchoscopy, the distal potion of tracheal tube can be mislocated accidentally and obstruct an anomalous bronchus. This condition may lead to right upper lobe atelectasis and hypoxemia.^[8]^

Nevertheless, the presence of a tracheal bronchus rarely affects the margin of safety of a left-sided double-lumen tube because the tracheal orifice of the double-lumen tube is much further from the carina than the tracheal bronchus.^[9]^

**Figure 3:**
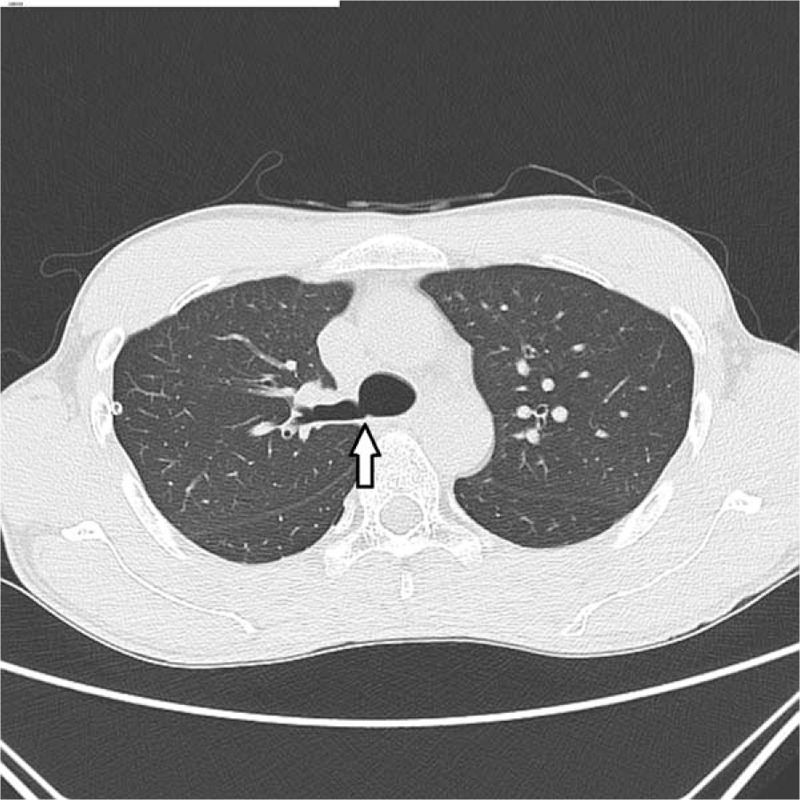
Preoperative axial chest computed tomography image shows acute angulation of the branching portion of the tracheal bronchus, mimicking the carina (arrow).

We tried 1-lung isolation with a left-sided double-lumen tube. In seeking to confirm the correct positioning of the tube, we observed what we thought was the carina by fiberoptic bronchoscopy, but it was, in fact, a site between the tracheal bronchus and the right main bronchus. In most cases, a tracheal bronchus is typically visible with the right and left main bronchi because of its anatomical characteristics, the tracheal orifice of the double-lumen tube is much further from the carina than the tracheal bronchus, as described above.^[9]^ However, in this case, we observed only the division of the right main bronchus and the tracheal bronchus at that level. Thus, a right-side intubation was performed incidentally, and the left main bronchus orifice was sealed by the bronchial cuff. As a result of inappropriate ventilation, PIP rose and SaO_2_ fell. It might have been better to take a screen capture of the bronchoscopic view, but usually this is not necessary in double-lumen tube intubation. The bronchoscopic view can also be assessed from axial chest CT images (Fig. 3).

In this case, it was important to detect the mislocation of the double lumen tube quickly. After induction, we confirmed the presence of the tracheal bronchus from a retrospective review of preoperative images. If we had reviewed the radiographs more carefully, we would have found the anatomical variation more readily and could have prepared for it in advance. Furthermore, we should have evaluated the right upper lobe bronchus distal to the carina when confirming the positioning of the left-sided double-lumen tube.

In conclusion, anesthesiologists should keep in mind the possibility of anatomical variation in the large airways, and bronchoscopy should be accompanied by careful auscultation of lung sounds with a stethoscope. Moreover, when performing fiberoptic bronchoscopy, we should confirm the position of the carina relative to the division of the bronchus carefully.
